# Supplementary material for: An in vitro tumorigenesis model based on live-cell-generated oxygen and nutrient gradients
Source: Commun Biol. 2021 Apr 15;4:477. doi: 10.1038/s42003-021-01954-0 (PMC8050328; doi:10.1038/s42003-021-01954-0)
Supplement: Supplementary file 2 — Supplementary Information [file 42003_2021_1954_MOESM2_ESM.pdf]

Supplementary information

An *in vitro* tumorigenesis model based on live-cell-generated oxygen and nutrient gradients

Anne C. Gilmore[1§], Sarah J. Flaherty[1], Veena Somasundaram[2], David A. Scheiblin[3], Stephen J. Lockett[3], David A. Wink[2], William F. Heinz[3*]

[1]Optical Microscopy and Analysis Laboratory, Office of Science and Technology Resources, Center for Cancer Research, National Cancer Institute, National Institutes of Health

[2]Laboratory of Cancer Immunometabolism, Center for Cancer Research, National Cancer Institute, National Institutes of Health

[3]Optical Microscopy and Analysis Laboratory, Cancer Research Technology Program, Frederick National Laboratory for Cancer Research, Frederick, MD

[§]Current address: Graduate School of Biomedical Sciences, St. Jude Children's Research Hospital, Memphis, TN.

Running title: Oxygen and nutrient gradient tumorigenesis model

Keywords: hypoxia, tumor microenvironment, live cell model

**Correspondence should be addressed to*:

William F. Heinz
Optical Microscopy and Analysis Laboratory
Frederick National Lab For Cancer Research
Leidos Biomedical Research, Inc.
P.O. Box B
Frederick, MD 21702
301-846-1239
will.heinz@nih.gov

Contents

	Page
Supplementary Figure 1	3
Supplementary Figure 2	4
Supplementary Figure 3	5
Supplementary Figure 4	7
Supplementary Figure 5	8
Supplementary Figure 6	9
Supplementary Figure 7	9
Supplementary Figure 8	10
Supplementary Figure 9	12
Supplementary Figure 10	13
Supplementary Figure 11	15
Supplementary Figure 12	17
Supplementary Figure 13	18
Supplementary Figure 14	19
Supplementary Method	21

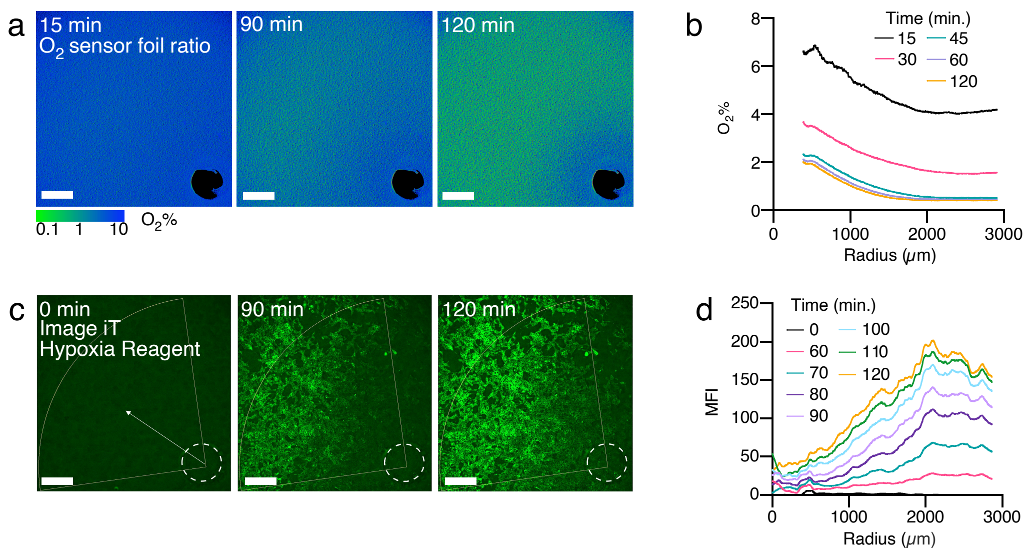

Supplementary Figure 1. Cell-generated [O_2] gradients in a 4T1-REEC

a. Cell-generated extracellular [O_2] gradients in a 4T1-REEC. Ratiometric images of an O_2 sensor foil over 2 hours after chamber placement on uniformly distributed 4T1 cells.

b. Radial quantification of extracellular [O_2] at various time points for the 4T1-REEC in (**a**).

c. Intracellular hypoxic gradients. Widefield images of Image IT Hypoxia Green Reagent (IHGR), which fluoresces in response to intracellular hypoxia, over two hours in a 4T1-REEC with uniformly distributed 4T1 cells. Dashed circles indicate the opening. Yellow circular sectors indicate the area used to calculate radial profiles. White arrow represents a radial value used to generate a radial profile.

d. Mean fluorescence intensity (MFI) per pixel of IGHR versus radial distance from opening at various time points.

Scale bars = 500 μm.

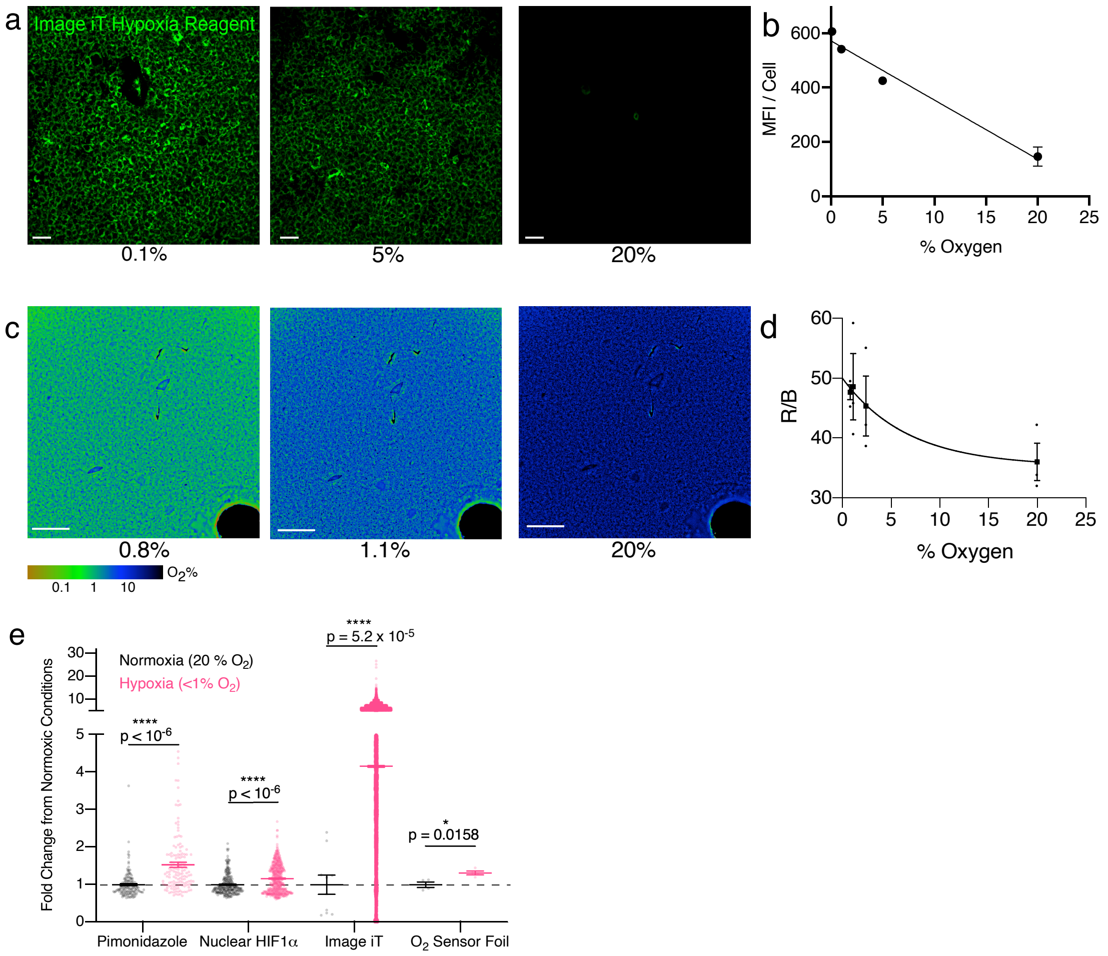

Supplementary Figure 2. Oxygen sensing techniques. Multiple complementary techniques were used to measure [O_2] and cellular hypoxia in conditions of spatially uniform [O_2] including a direct measure of dissolved [O_2] and live-cell and end-point probes of intracellular hypoxia. All methods respond to [O_2] < 1% in the REEC system.

a. Widefield images of Image IT Green Hypoxia Reagent (IGHR) response in 4T1 cells to hypoxia. Scale bar = 50 µm.

b. Calibration curve of IGHR mean fluorescence intensity per cell to measured oxygen concentration (0.1%, N = 13,214; 1%, N = 27,628; 5%, N = 8,815, 20%, N = 10. N = cell number) showing an approximately linear decrease. Note that at 20% O_2, few cells had measurable fluorescence.

c. Ratiometric images of PreSens O_2-sensing foil response to [O_2] levels in glucose oxidase / catalase solutions of known [O_2]. Scale bar = 500 µm.

d. Calibration curve of O_2-sensing foil ratiometric response in control glucose-oxidase solutions showing an approximately exponential decay (N = 3).

e. Quantification of fold change in fluorescent signal compared to normoxic conditions of pimonidazole, nuclear-localized HIF1α, IGHR, and PreSens O_2-sensing foils O_2 in response to low oxygen (<1%) (N > 100 4T1 pimonidazole, nuclear-localized HIF1α, IGHR; N = 4 for O_2 foils).

Data plotted as mean ± SEM.

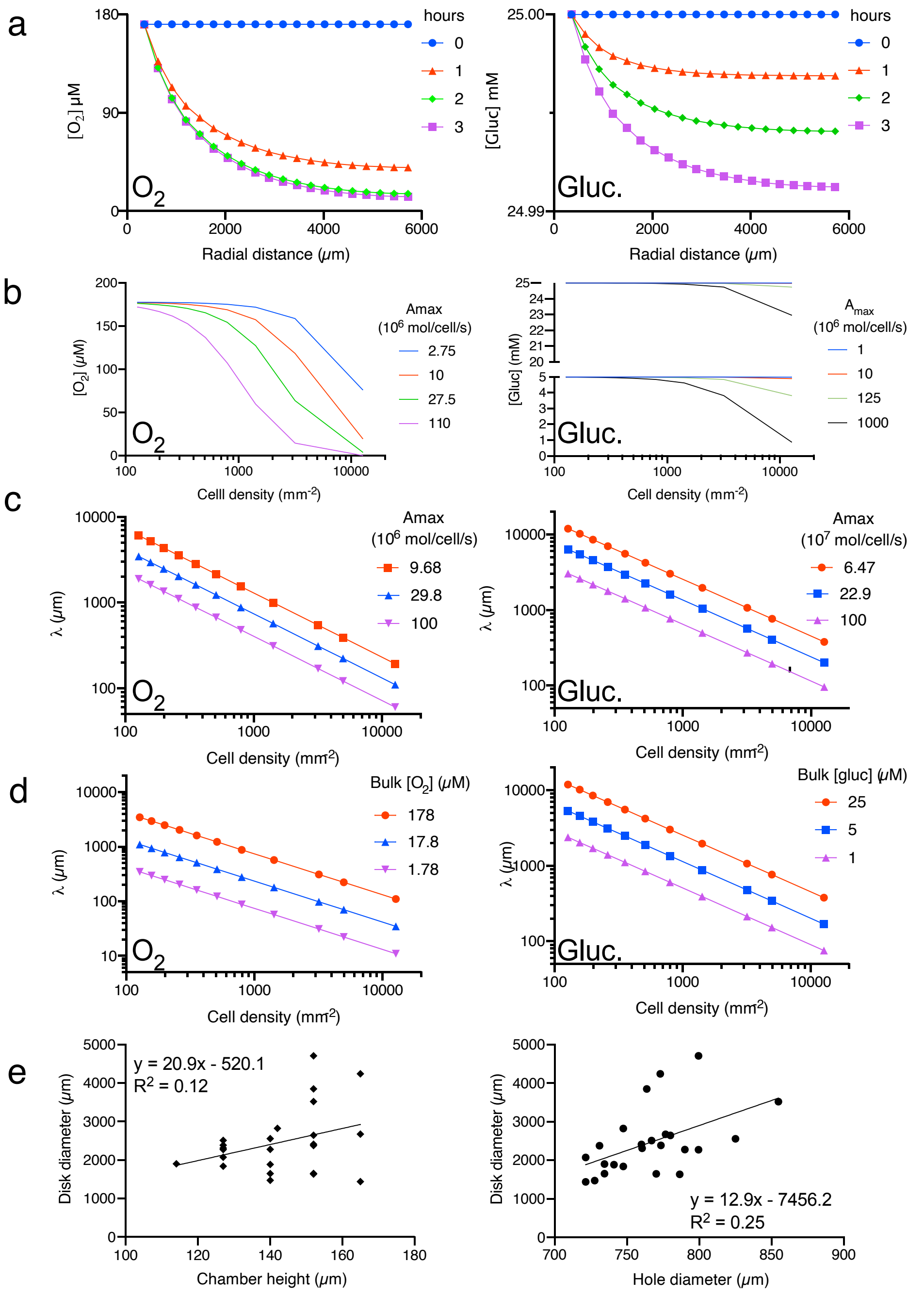

Supplementary Figure 3. Diffusion-consumption 2D in silico model captures the gradient dynamics and steady state of the 4T1-REEC system.

a. Time evolution of the radial profiles of the O_2 (left) and glucose (right) concentrations (30-minute time steps) calculated with the chamber dimensions (hole diameter = 700 µm), 4T1 consumption rates (2.98×10^7 and 6.47×10^7 molecules/cell/s for O_2 and glucose, respectively), cell densities (cell diameter = 13.5 µm), and bulk concentrations used *in vitro* ($[O_2]$ = 171 µM, [Gluc.] = 25 mM). The $[O_2]$ decreases to a stable gradient within 2 hours. The glucose concentration changes little because the bulk concentration, 25 mM, is in excess of the cells' metabolic demands.

b. O_2 (left) and glucose (right) concentrations as a function of cell density and maximum consumption rate in normoxic nutrient-rich media, A_{max}, after 48 hours. Glucose concentration curves are shown for bulk concentrations of 25 mM (top) and 5 mM (bottom).

c. Characteristic steady-state distance, $\lambda = \sqrt{DC_{bulk}/nA_{max}}$, as a function of cell density, *n*, and maximum consumption rate, A_{max}. *D* is diffusion coefficient. λ is related to the stable cell disk radius. This estimation is independent of REEC geometry. Bulk concentrations (C_{bulk}) were 178 µM and 25 mM for O_2 (left) and glucose (right), respectively.

d. Characteristic steady-state distance as a function of cell density and bulk concentration. Here, A_{max} values for 4T1 cells were 2.98×10^7 and 6.47×10^7 molecules/cell/s for O_2 (left) and glucose (right), respectively.

e. Empirical measurements of 4T1 cell disk size depends on chamber height and hole diameter.

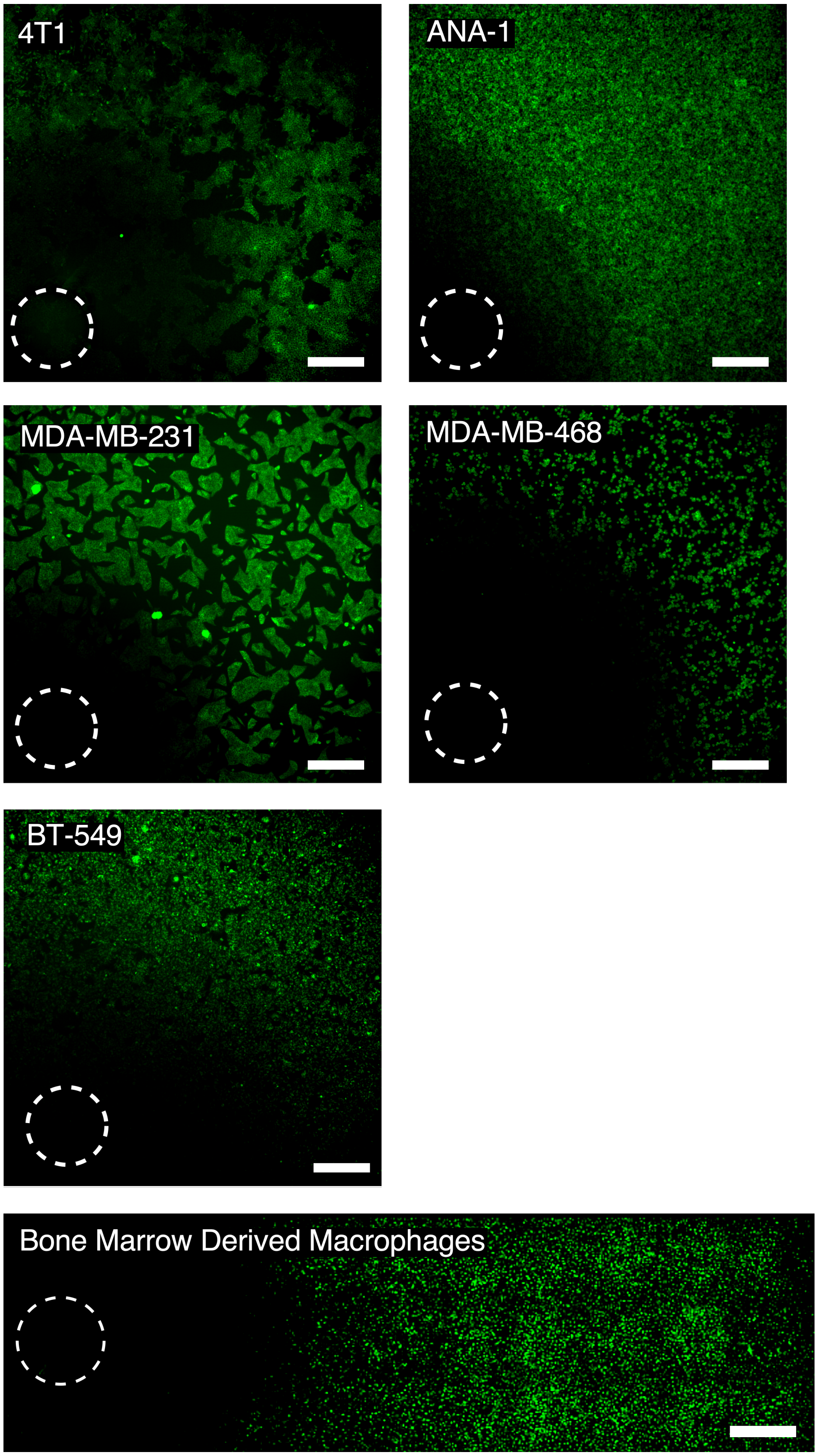

Supplementary Figure 4. Human TNBC cells and mouse macrophages generate hypoxic gradients in REECs. Widefield images of mouse 4T1 cells, human TNBC cells (MDA-MB-231, MDA-MB-468, and BT-549), mouse macrophages (ANA-1 and bone marrow-derived machrophages) stained with Image IT Green Hypoxia Reagent (IGHR) reveal hypoxic gradients in REECs 2 hours after chamber placement. The cells were uniformly present in the chambers, but only hypoxic cells fluoresce. The variation in distance to the hypoxic front likely results from differential oxygen consumption rates of the cell lines. Dashed circle indicates the opening. Scale bars = 500 µm.

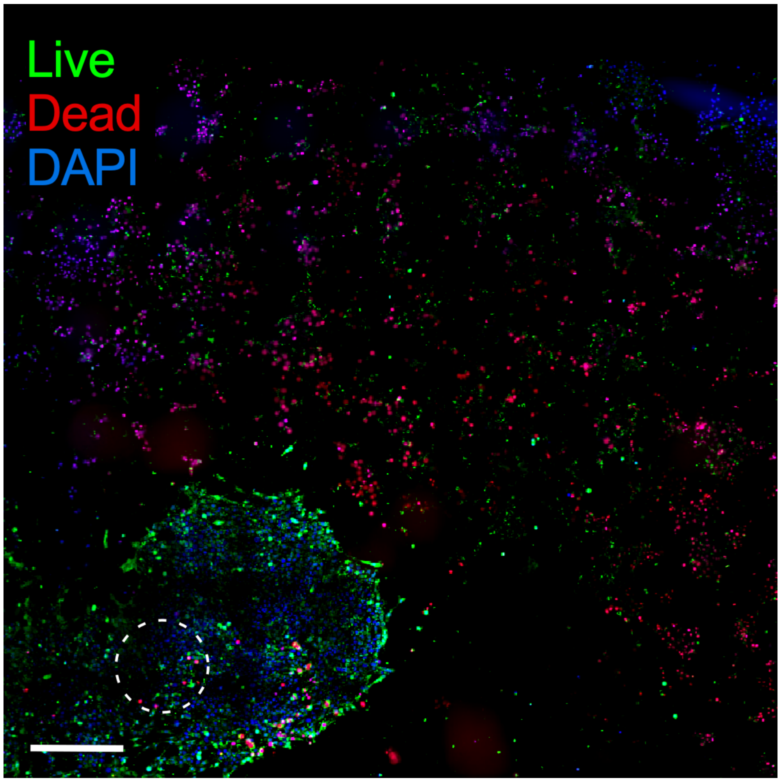

Supplementary Figure 5. Cell viability across the REEC. Live/dead staining shows necrotic zone beyond the cell disk live/dead staining in 4-day old 4T1 disks. Live cells are stained by calcein AM (green). Dead cells are stained by ethidium homodimer (red). Dashed circle indicates the opening. Scale bar = 500 μm.

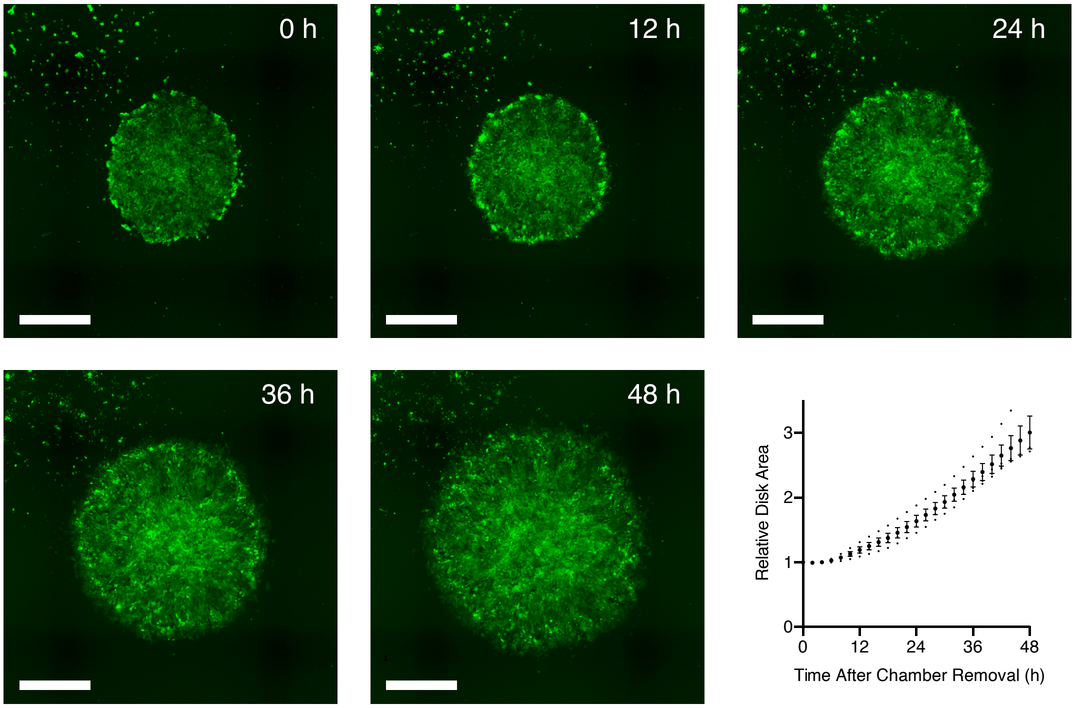

Supplementary Figure 6. 4T1 proliferation is restored after the REEC is removed and normoxia is established across the disk. After disk formation (92 hours after placement of the chamber top), the chamber top was removed and 4T1 growth was monitored over 48 hours. Data plotted as mean ± SEM (N = 4 disks). Scale bar = 200 µm.

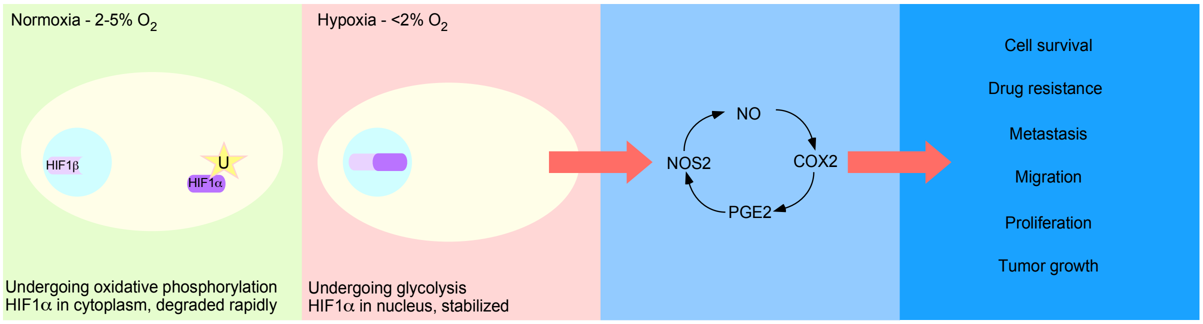

Supplementary Figure 7. Cellular Response to Hypoxia. In normoxic conditions HIF1α is rapidly ubiquinated and degraded in the cytoplasm, and HIF1β localizes to the nucleus (left). In hypoxic conditions HIF1α localizes to the nucleus to form a stable dimer with HIF1β (left center). This leads to cell survival and a tumorigenic phenotype, as well as upregulation of the inflammatory protein NOS2 (right center). NOS2 and COX2 engage in a feedforward loop via nitric oxide (NO) and prostaglandin E2 (PGE2) signaling. NOS2 expression can promote a pro-tumor phenotype (right).

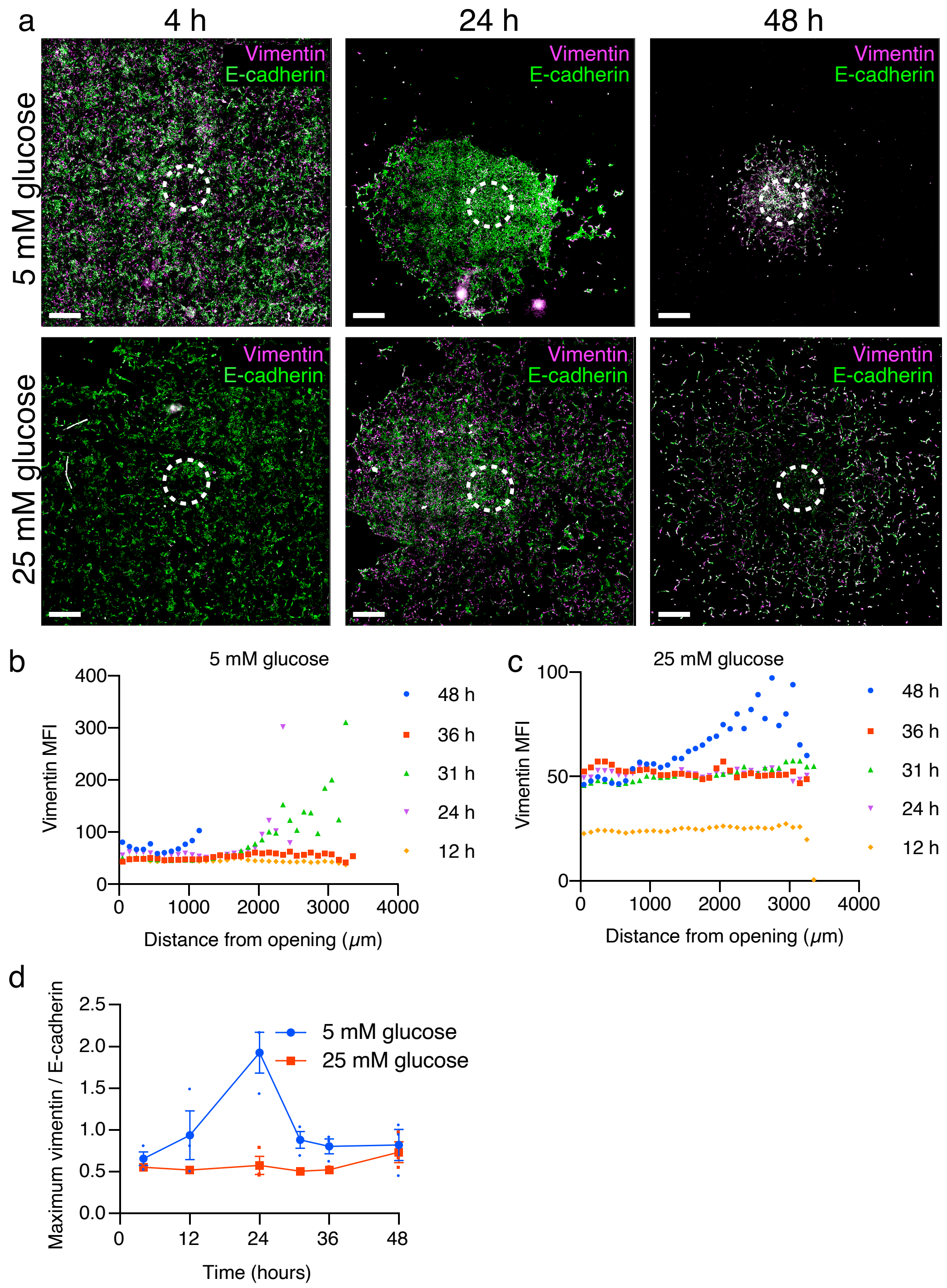

Supplementary Figure 8. Epithelial mesenchymal transition (EMT) marker expression depends on glucose concentration and time.

a. Widefield images of vimentin and E-cadherin immunofluorescence staining of 4T1 cells cultured in REECs in low (5 mM) or high (25 mM) glucose media at various timepoints after chamber placement. In these images, white pixels result from the overlap of E-cadherin (green) and vimentin (magenta) signals. The checkered board pattern is a technical artefact due to tiling of images. Scale bar = 500 µm.

- **b.** Expression of vimentin in low glucose (5 mM) media as a function of distance from opening at different time points (N = 1 per timepoint). Vimentin expression is transiently increased in the hypoxic regions of the disk from 24 to 31 hours during disk formation. MFI = mean fluorescence intensity.
- **c.** Expression of vimentin in high glucose (25 mM) media as a function of distance from opening at different time points (N = 1 per timepoint). The 12 h timepoint is low because vimentin is an endpoint EMT marker, and at 12 h EMT has not yet begun. At later times the vimentin expression is elevated. MFI = mean fluorescence intensity.
- **d.** Ratio of maximum vimentin to E-cadherin expression in disks for different glucose concentrations over time (N = 3 disks, 5 mM glucose; N = 2 disks, 25 mM glucose). The E-cadherin levels did not vary appreciably across the REEC over time. In the 5 mM case, after peaking during disk formation, the ratio returns to the initial value in stable disks. Data plotted as mean $\pm$ SEM.

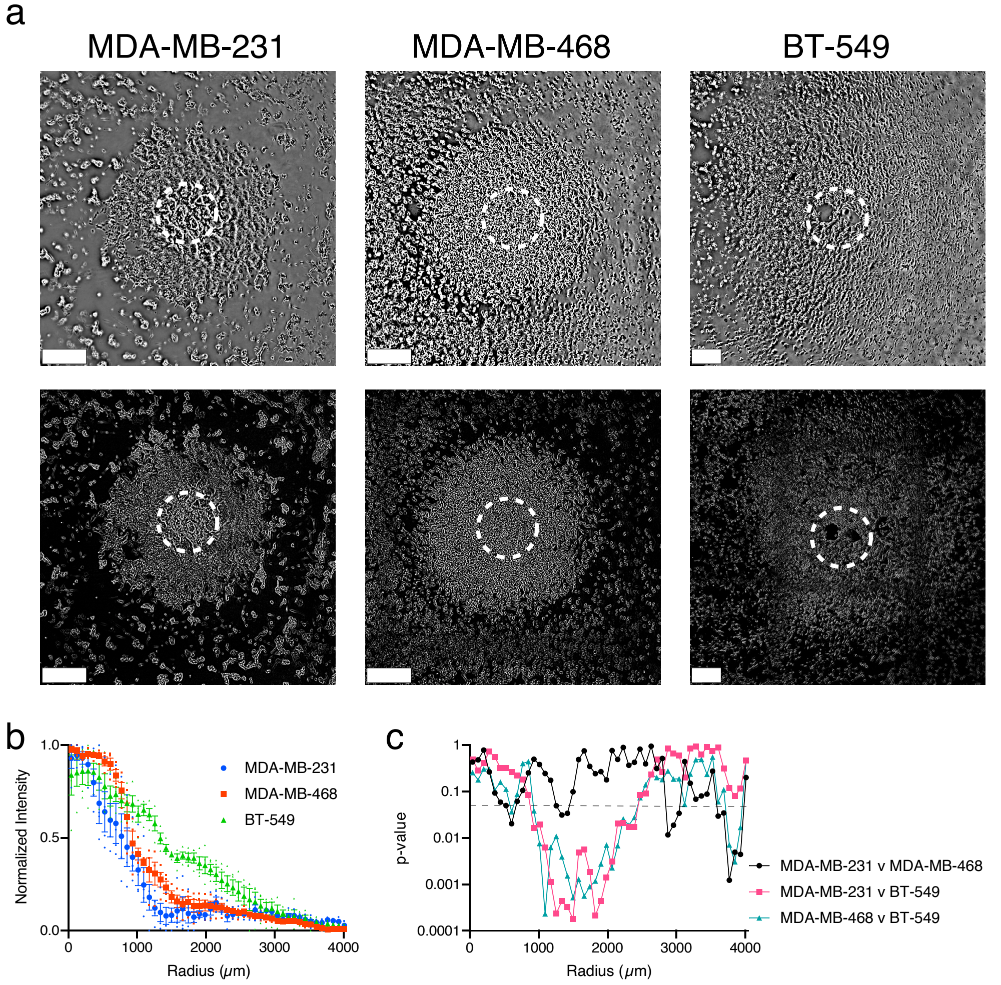

d

Cell line	Disk formation time (h)	N
MDA-MB-231	68 ± 1.3	3
MDA-MB-468	106.7 ± 2.0	3
BT-549	135 ± 0.7	2

Supplementary Figure 9. Human TNBC cells form disks in REECs.

a. Bandpass-filtered (top) and background-subtracted (bottom) phase images of MDA-MB-231, MDA-MB-468, and BT-549 7 day-old cell disks in REECs. Dashed circle indicates the opening. Scale bars = 500 µm.

b. Radial intensity profiles of the background-subtracted phase images of the MDA-MB-231, MDA-MB-468, and BT-549 7-day old cell disks in REECs (N = 4 per cell type). Data displayed as mean ± SEM.

c. P-values between radial profiles of the different cell types at each radius. The dashed line represents p = 0.05.

d. Disk formation times for the three human cell lines in REECs.

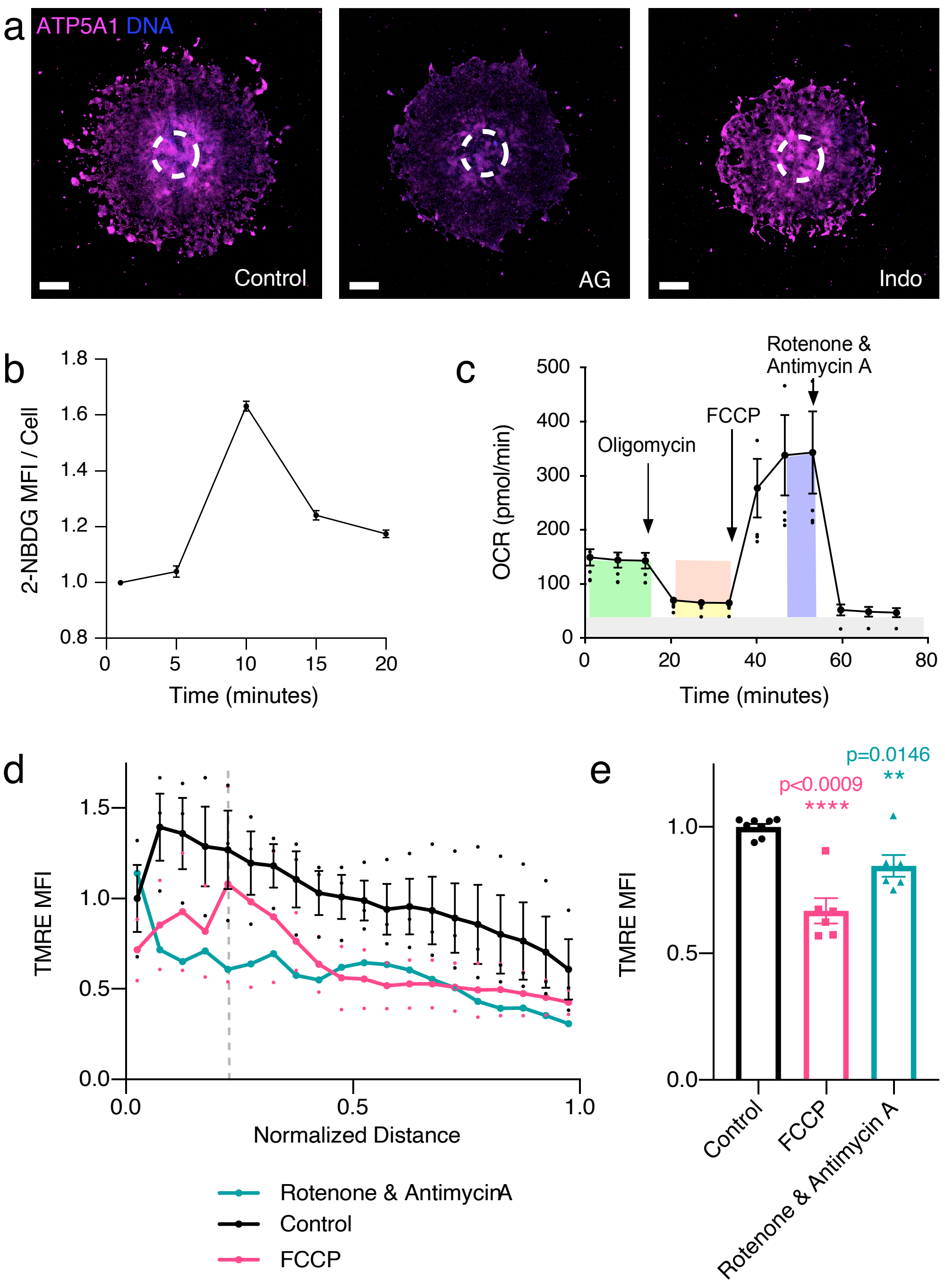

Supplementary Figure 10. Mitochondrial controls.

a. Widefield images of ATP5A, a mitochondrial ATPase, used here as a measure of mitochondrial mass, immunofluorescence staining in 7-day old 4T1 disks, including a control disk (left), a disk treated with aminoguanidine (middle), and a disk treated with indomethacin (right). Scale bar = 500 µm.

b. 2-NBDG uptake in 4T1 cells over time in standard culture vessel (N > 135 cells). Data plotted as mean ± SEM.

- **c.** 4T1 oxygen consumption rate (OCR) measured by Seahorse Mito Stress test (N = 4 wells). Yellow represents basal respiration, green represents ATP-linked respiration, orange represents proton leak, purple represents maximal respiration, and grey represents non-mitochondrial contributions to OCR, respectively. Data plotted as mean $\pm$ SEM.
- **d.** Quantification of TMRE response (mean fluorescence intensity normalized to first point of the untreated control) across 4T1 disks before and after treatment with rotenone and antimycin-A or FCCP (N= 3, 2, 1 disks for control, FFCP, and rotenone & antimycin A, respectively). Vertical dashed line represents the edge of the opening. Images were collected 20 minutes after treatments. Data plotted as mean $\pm$ SEM.
- **e.** Quantification of TMRE response (normalized to the untreated control) before and after treatment with antimycin-A and rotenone or FCCP before and after treatments in standard cell culture conditions (N= 8, 6, 6 wells for control, FFCP, and rotenone & antimycin A, respectively). Data plotted as mean $\pm$ SEM.

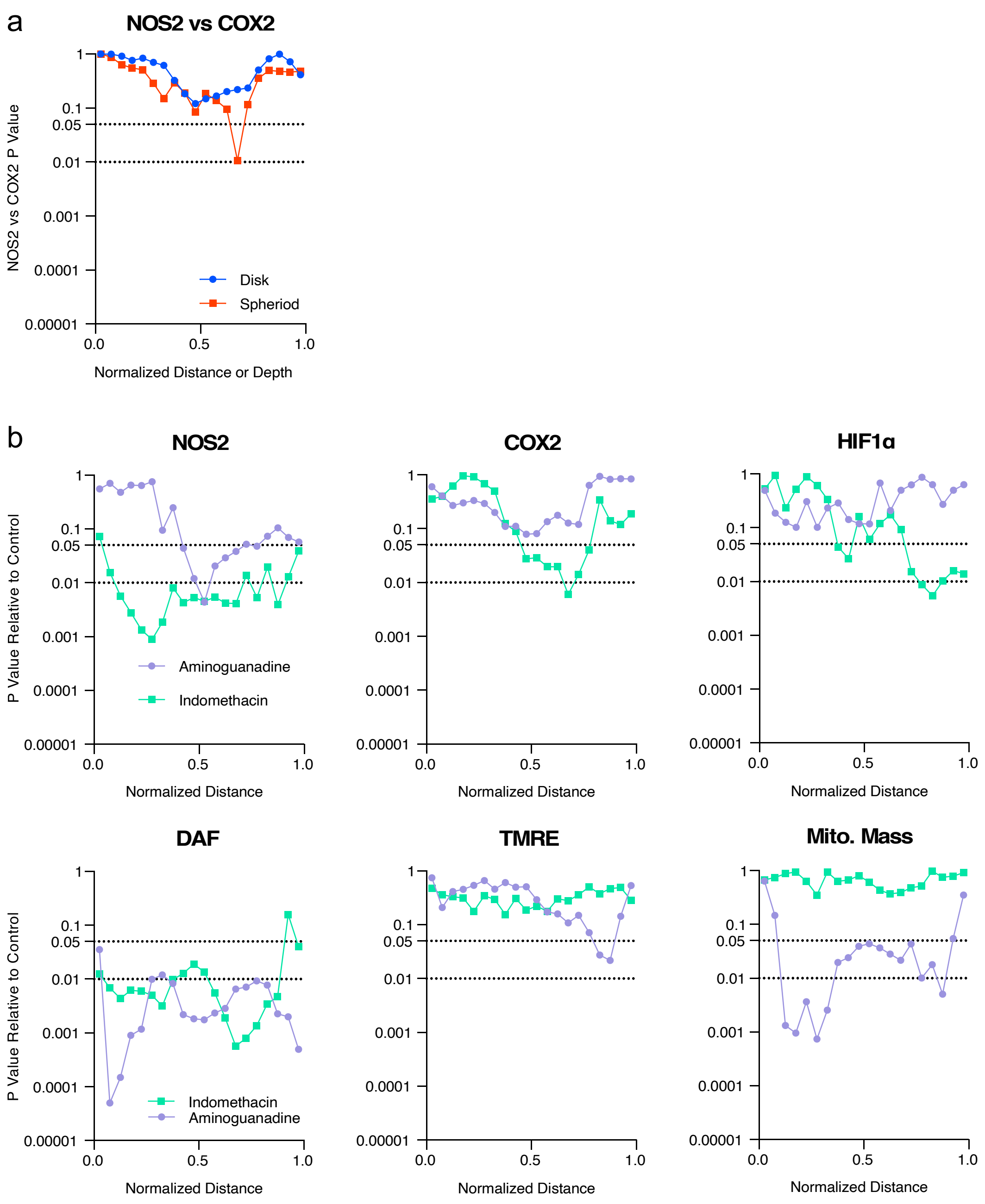

Supplementary Figure 11 Statistics supplement for Figures 3 and 4. P-Values were calculated at the normalized distances or depths corresponding to data points in Figures 3 and 4.

a. P-values quantifying the differences between the NOS2 and COX2 MFI/cell values in the control disks and spheroids that were presented in Fig. 3. Note that the NOS2 and COX2 signals are most different from one another in the mid-hypoxic region of both disks and spheroids (Normalized distance or depth between 0.5 and 0.75).

b. P-values quantifying the differences between the MFI/cell values observed in the control disks and the MFI/cell values observed in the aminoguanidine- or indomethacin-treated disks that were presented in Fig. 4. The p-values were calculated using the MFI/cell of NOS2, COX2, HIF1α, DAF, TMRE, and mitochondrial mass (measured by ATP5A).

"Normalized Distance" refers to the relative distance from the center to the edge of a disk.
"Normalized Depth" refers to relative distance from the surface to the center of a spheroid.
MFI = mean fluorescence intensity normalized to the first point.

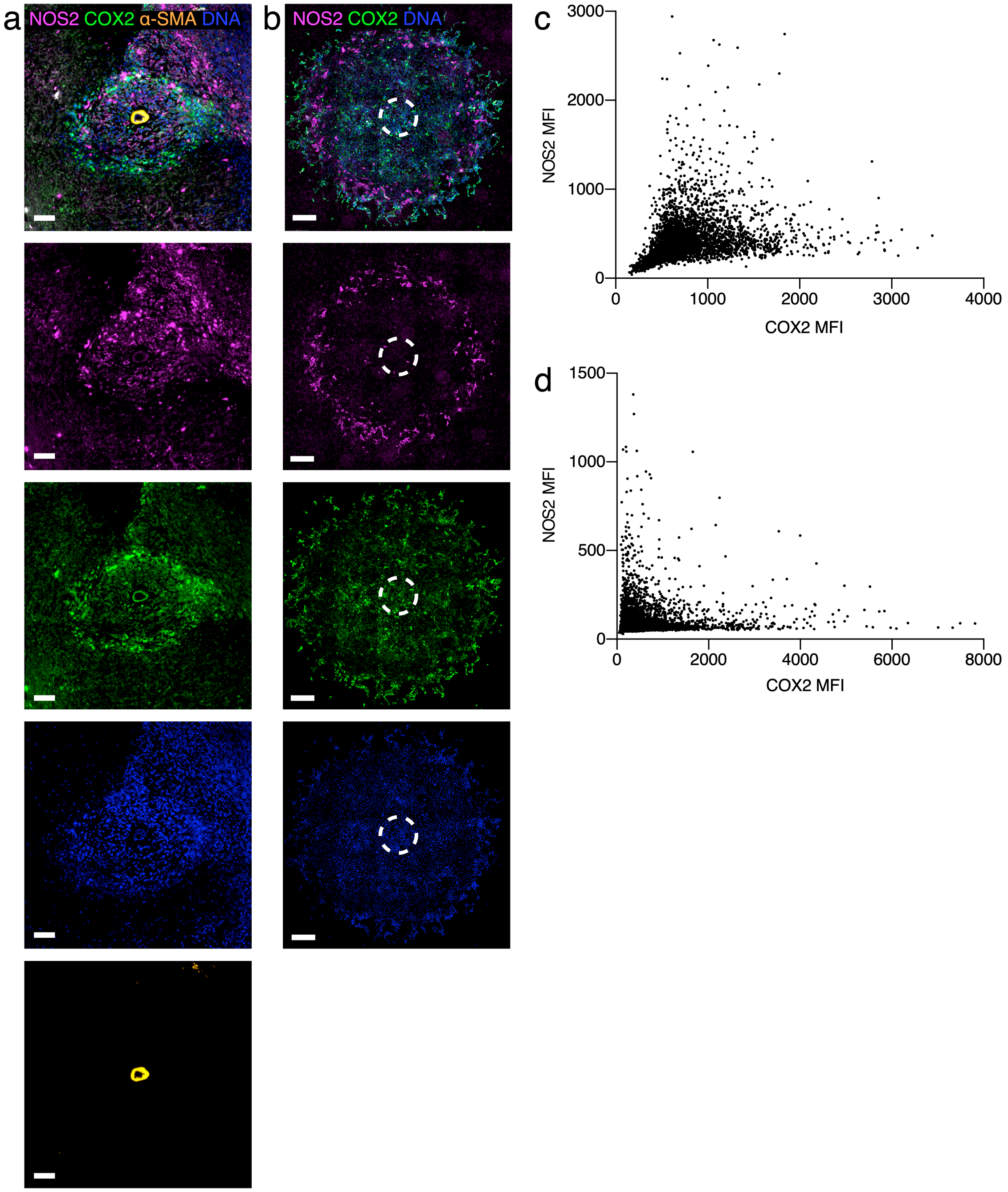

Supplementary Figure 12. NOS2 and COX2 are expressed in different cells.

a. Widefield images of NOS2, COX2, α-SMA, and DAPI multiplexed immunofluorescence staining surrounding a blood vessel in a 4T1 tumor. Scale bar = 500 µm.

b. Widefield images of NOS2, COX2, and DAPI immunofluorescence staining of a 7-day old 4T1 disk. Scale bar = 500 µm.

c. NOS2 vs. COX2 intensity for each cell in the 4T1 tumor section shown in **a** (N = 4196; Pearson's correlation coefficient ρ=0.2477).

d. NOS2 vs. COX2 intensity for each cell in the 4T1 disk shown in **b** (N = 7339; Pearson's correlation coefficient ρ=0.0969).

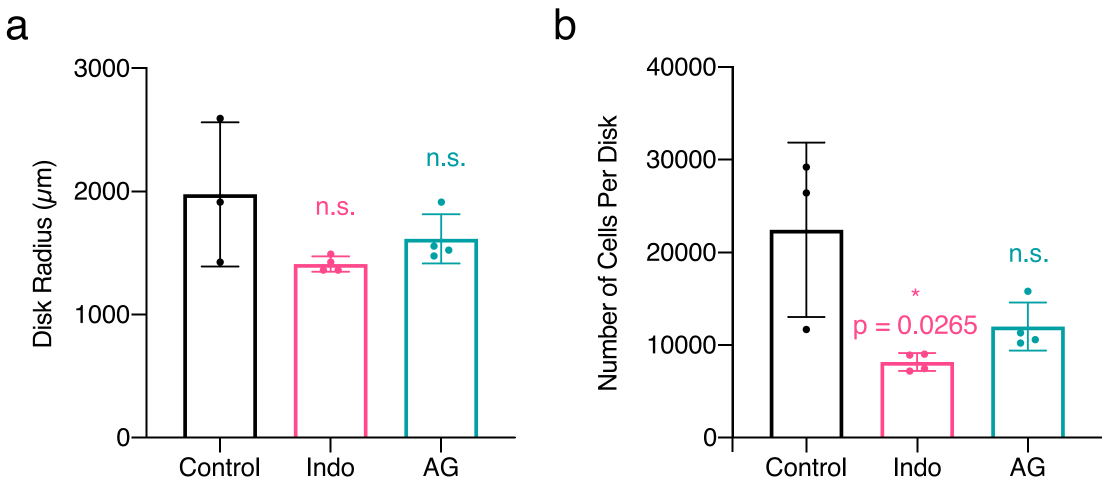

Supplementary Figure 13. Effect of treatment on 4T1 disk size. 4T1 disks treated with indomethacin have significantly fewer cells than control 4T1 disks, but there is no significant difference in 4T1 disk radius after Indo or AG treatments.

a. Average radius of control disks and disks treated with indomethacin or aminoguanidine.

b. Average number of cells per disk in control disks and disks treated with indomethacin or aminoguanidine.

N = 3 control disks; N = 4 Indo treated disks; N = 4 AG treated disks. Data plotted as mean ± SEM.

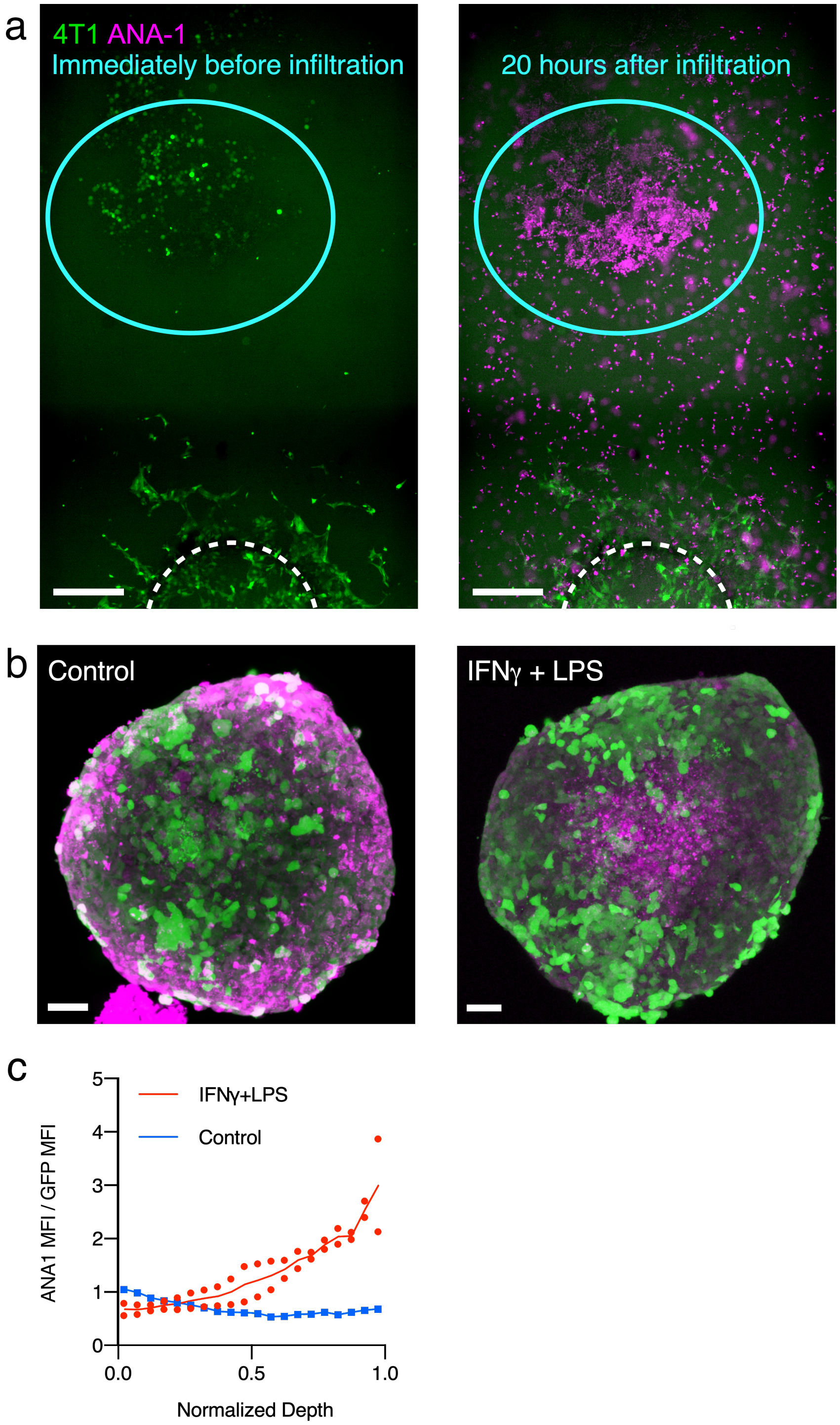

Supplementary Figure Supplementary Figure 14. ANA-1 macrophages survive in the hypoxic and necrotic zones of 4T1 disks, and stimulated ANA-1 macrophages infiltrate the hypoxic core of spheroids.

a. After 4 days, GFP-4T1 cells (green) form a disk, while some dying GFP-4T1s (outlined with blue dashed circle) remain in the hypoxic region of the chamber, far from the opening (left). ANA-1 macrophages were injected into the chamber through the hole (dashed half-circle). Within 20 hours after macrophage (magenta) injection, the macrophages are localized to the necrotic zone (right). Scale bar = 700 µm.

b. Confocal microscope images of the equatorial planes of cleared GFP-4T1 spheroids with unstimulated (left) ANA-1 macrophages (magenta) and IFNγ + LPS stimulated macrophages (right) demonstrate stimulated macrophages infiltrate the hypoxic core of 4T1 (green) spheroids. Scale bar = 50 µm.

c. Quantification of the ratio of ANA-1 mean fluorescent intensity to GFP mean fluorescent intensity as a function of depth from the surface to the center of the spheroids (N = 2 per group). Data plotted with mean (line).

Supplementary Method

REEC construction and use

Materials

- Compressed air drill (400x High Speed Engraver, SCM Systems, Menomonee Falls, WI)
- UV Curable epoxy (NOA81, Norland Products, Cranbury, NJ)
- UVO-Cleaner Model 342 (Jelight Company Inc., Irvine, CA)
- 100µm tall 18 mm stainless steel O-rings (PN 90214A123, McMaster-Carr, Robbinsville, NJ)
- 18 mm diameter coverslips, number 1 thickness
- Glass slides
- Scotch tape
- Ethanol
- Forceps (Ted Pella, Redding, CA)
- 500 µm drill bits (SCM Systems)
- 12-well glass bottom plates (PN P12-1.5H-N, CellVis, Mountain View, CA)
- Laser cut mylar clamps
- Laser cut mylar 12-well plate rims

Warnings

Make sure to wear appropriate personal protective equipment, including eye and ear protection during glass drilling.

Making chambers and plates

Modify the multiwell plate

1. Apply UV curable epoxy carefully around each well on a 12-well, glass-bottom plate
2. Apply rims so that each well has even overhang of mylar
3. Cure in UV box for five minutes
4. Use immediately for cell culture or store somewhere covered and protected, sterilize in UV box for at least three minutes before use

Assemble the REEC

1. Tape a 18 mm coverslip to a clean glass slide to prevent the coverslip from moving during the drilling. Tape applied to opposite edges of the coverslip, or to 3 equally space positions around the edge is usually sufficient.
2. Drill a hole through the center of the coverslip. Hold the drill normal to the glass surface and press lightly with the drill on the glass -- do not push hard.
3. Once the drill has gone through the coverslip, remove the coverslip from the slide. Clean debris from the coverslip with kimwipes and ethanol. It is important to ensure there are no pieces of glass or tape residue on the coverslip.
4. Apply UV curable epoxy carefully to a stainless-steel O-ring, place glass coverslip using forceps on the O-ring. Avoid applying too much epoxy.
5. Cure in UVO-Cleaner for five minutes, glass side up.
6. Apply glue around clamp

7. Place glued coverslip and O-ring on the clamp so that the glass is in between the mylar clamp and the stainless-steel O-ring
8. Cure in UVO-Cleaner for five minutes, mylar side up.
9. Flip over with forceps
10. Place in UVO-Cleaner for three minutes.
11. At this point, the chambers are sterile. Use immediately or store in covered location and sterilize in UVO-Cleaner for at least 3 minutes, each side, before use.

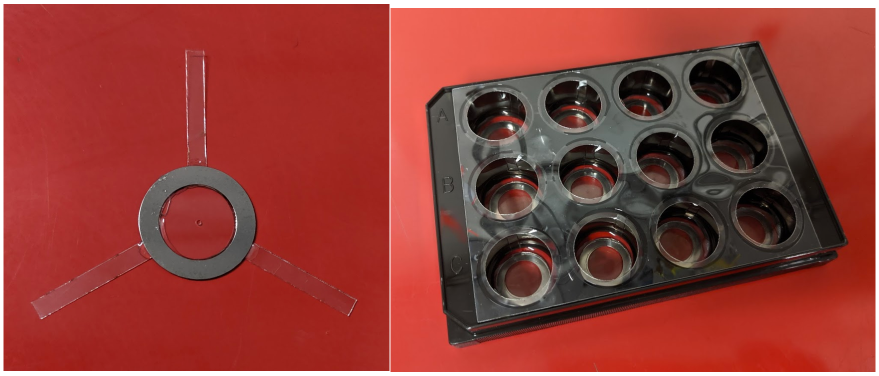

REEC assembly. Left: Fully assembled REEC, including coverslip with through hole, mylar clamps and O-ring. **Right:** Fully assembled REECs in a well of a 12-well plate with PBS.

Cell Culture for REECs

1. Plate 1 mL of cells per well with desired density of cells in sterile, rim-glued, 12-well, glass-bottom plate. For 4T1s, use a density of 400,000 cells/mL.
2. Allow cells to adhere for an appropriate time. This will vary by cell type. For 4T1s, allow to adhere overnight.

To place chamber, use forceps to gently push sterilized chamber down onto cells. Avoid scratching surface and removing cells or allowing air bubbles to form in the chamber.
