# Supplementary material for: An in vitro tumorigenesis model based on live-cell-generated oxygen and nutrient gradients
Source: Commun Biol. 2021 Apr 15;4:477. doi: 10.1038/s42003-021-01954-0 (PMC8050328; doi:10.1038/s42003-021-01954-0)
Supplement: Supplementary file 3 — Description of Additional Supplementary Files [file 42003_2021_1954_MOESM3_ESM.pdf]

Description of Additional Supplementary Files

File Name: Supplementary Movie 1

Description: 4T1 cells migrate up gradient to form confluent disk. GFP-expressing 4T1 cells imaged every 2 hours at 4x magnification in the REEC. Disk forms after about 60 hours. The white circle represents the REEC opening.

File Name: Supplementary Movie 2

Description: The 4T1 cell disk expands by migration and proliferation after chamber removal. GFP-expressing 4T1 cells imaged every 2 hours at 4x magnification in the REEC after chamber top removal. The center of the disk is in the upper left corner, only one quadrant of the disk is shown. Arrows point to migrating cells that divide.

File Name: Supplementary Data 1

Description: All data shown in the quantitative plots.
