# Supplementary material for: An in vitro tumorigenesis model based on live-cell-generated oxygen and nutrient gradients
Source: Commun Biol. 2021 Apr 15;4:477. doi: 10.1038/s42003-021-01954-0 (PMC8050328; doi:10.1038/s42003-021-01954-0)
Supplement: Supplementary file 7 — Reporting Summary [file 42003_2021_1954_MOESM7_ESM.pdf]

nature research

Corresponding author(s): William Heinz

Last updated by author(s): Feb 20, 2021

Reporting Summary

Nature Research wishes to improve the reproducibility of the work that we publish. This form provides structure for consistency and transparency in reporting. For further information on Nature Research policies, see our Editorial Policies and the Editorial Policy Checklist.

Statistics

For all statistical analyses, confirm that the following items are present in the figure legend, table legend, main text, or Methods section.

n/a	Confirmed	
☐	☒	The exact sample size (*n*) for each experimental group/condition, given as a discrete number and unit of measurement
☐	☒	A statement on whether measurements were taken from distinct samples or whether the same sample was measured repeatedly
☐	☒	The statistical test(s) used AND whether they are one- or two-sided<br>*Only common tests should be described solely by name; describe more complex techniques in the Methods section.*
☒	☐	A description of all covariates tested
☒	☐	A description of any assumptions or corrections, such as tests of normality and adjustment for multiple comparisons
☐	☒	A full description of the statistical parameters including central tendency (e.g. means) or other basic estimates (e.g. regression coefficient) AND variation (e.g. standard deviation) or associated estimates of uncertainty (e.g. confidence intervals)
☒	☐	For null hypothesis testing, the test statistic (e.g. *F*, *t*, *r*) with confidence intervals, effect sizes, degrees of freedom and *P* value noted<br>*Give P values as exact values whenever suitable.*
☒	☐	For Bayesian analysis, information on the choice of priors and Markov chain Monte Carlo settings
☒	☐	For hierarchical and complex designs, identification of the appropriate level for tests and full reporting of outcomes
☐	☒	Estimates of effect sizes (e.g. Cohen's *d*, Pearson's *r*), indicating how they were calculated

Our web collection on statistics for biologists contains articles on many of the points above.

Software and code

Policy information about availability of computer code

Data collection	Image data was collected using Nikon Elements v 5.02., Zeiss Zen Black v 2.3, Leica LSX SP8. O2 data for calibrations was collected using PyroScience GmbH Pyro Oxygen Logger 3.2. OCR data was collected using Agilent Seahorse Wave Desktop v 2.6.
Data analysis	Image data was processed and analyzed using FIJI v 2.00rc 69/1.52p, Imaris v 9.5.1, MATLAB 2019b, Excel v 15.36, and R v 1.2.1335.

For manuscripts utilizing custom algorithms or software that are central to the research but not yet described in published literature, software must be made available to editors and reviewers. We strongly encourage code deposition in a community repository (e.g. GitHub). See the Nature Research guidelines for submitting code & software for further information.

Data

Policy information about availability of data

All manuscripts must include a data availability statement. This statement should provide the following information, where applicable:

- Accession codes, unique identifiers, or web links for publicly available datasets
- A list of figures that have associated raw data
- A description of any restrictions on data availability

The data that support the findings of this study are available from the corresponding author upon request.

Field-specific reporting

Life sciences study design

All studies must disclose on these points even when the disclosure is negative.

Sample size	Measurements of individual cell disks quantified the fluorescence intensity and position of ~104 cells per disk, and data from multiple (e.g., N ≥ 3) disks per experimental condition were averaged for each analysis.
Data exclusions	No data were excluded from the analyses.
Replication	Replicate experiments were successful.
Randomization	Not applicable to this study
Blinding	Not applicable to this study.

Reporting for specific materials, systems and methods

We require information from authors about some types of materials, experimental systems and methods used in many studies. Here, indicate whether each material, system or method listed is relevant to your study. If you are not sure if a list item applies to your research, read the appropriate section before selecting a response.

Materials & experimental systems

n/a	Involved in the study	
☐	☒	Antibodies
☐	☒	Eukaryotic cell lines
☒	☐	Palaeontology and archaeology
☐	☒	Animals and other organisms
☒	☐	Human research participants
☒	☐	Clinical data
☒	☐	Dual use research of concern

Methods

n/a	Involved in the study	
☒	☐	ChIP-seq
☒	☐	Flow cytometry
☒	☐	MRI-based neuroimaging

Antibodies

Antibodies used	E-cadherin, direct conjugate (AlexaFluor-488), P/N 3199S, Clone 24E10, Cell Signaling Technologies.<br>Vimentin, direct conjugate (AlexaFluor-555), P/N 9855S, Clone D21H3, Cell Signaling Technologies.<br>Nos2, direct conjugate (AlexaFluor-568), P/N 209595, Clone EPR16635, AbCam.<br>Cox2, direct conjugate (AlexaFluor-488), P/N 13596S, Clone D5H5, AbCam.<br>HIF1a, direct conjugate (AlexaFluor-647), P/N 208420, Clone EPR16897, AbCam.<br>ATP5A1 (Mitochondrial ATPase, polyclonal Primary), P/N A5884, AbClonal.<br>Anti-rabbit CF-640R (polyclonal secondary for ATPase), P/N 20178, BioTium.
Validation	Antibody validation data can be found at manufacturers' websites.

Eukaryotic cell lines

Policy information about cell lines

Cell line source(s)	4T1 cells, ATCC; 4T1-Fluc-Neo/eGFP-Puro cells, Imanis Life Sciences; ANA-1 cells established at Varesio Laboratory at NCI-Frederick. MDA-MB-231, MDA-MB-468, and BT-549 cells, Division of Cancer Treatment and Diagnosis (DCTD) Tumor Repository, Biological Testing Branch at the National Cancer Institute. Balb/c mouse BMDMs, Wink Laboratory at NCI-Frederick.
Authentication	The 4T1 and 4T1-Fluc-Neo/eGFP-Puro cells were validated by vendors. The MDA-MB-231, MDA-MB-468, and BT-549 cells were authenticated by DCTD and the Protein Expression Laboratory and RAS Reagents Core, Frederick National Laboratory for Cancer Research. Balb/C mouse BMDMs, Wink Laboratory at NCI-Frederick.
Mycoplasma contamination	All cell lines tested negative for mycoplasma contamination.
Commonly misidentified lines<br>(See ICLAC register)	No commonly misidentified cell lines were used in this study.

Animals and other organisms

Policy information about studies involving animals; ARRIVE guidelines recommended for reporting animal research

Laboratory animals	Female BALB/c mice, aged 8-10 weeks old, were obtained from the Frederick Cancer Research and Development Center Animal Production Area.
Wild animals	The study did not involve wild animals.
Field-collected samples	The study did not involve field-collected samples.
Ethics oversight	NCI-Frederick is accredited by AAALAC International and follows the Public Health Service Policy for the Care and Use of Laboratory Animals. Animal care was provided in accordance with the procedures outlined in the "Guide for Care and Use of Laboratory Animals" (National Research Council; 2011; National Academy Press; Washington, D.C.).

Note that full information on the approval of the study protocol must also be provided in the manuscript.
